# Shiga Toxin 2-Induced Endoplasmic Reticulum Stress Is Minimized by Activated Protein C but Does Not Correlate with Lethal Kidney Injury

**DOI:** 10.3390/toxins7010170

**Published:** 2015-01-20

**Authors:** Caitlin S. L. Parello, Chad L. Mayer, Benjamin C. Lee, Amanda Motomochi, Shinichiro Kurosawa, Deborah J. Stearns-Kurosawa

**Affiliations:** Department of Pathology and Laboratory Medicine, Boston University School of Medicine, 670 Albany Street, Boston, MA 02118, USA; E-Mails: csleibow@bu.edu (C.S.L.P.); cmayer@bu.edu (C.L.M.); bclee@bu.edu (B.C.L.); amoto@bu.edu (A.M.); dstearns@bu.edu (D.J.S.-K.)

**Keywords:** Shiga toxin, endoplasmic reticulum stress, apoptosis, kidney injury, enterohemorrhagic *Escherichia coli*, hemolytic uremic syndrome

## Abstract

Enterohemorrhagic *Escherichia coli* produce ribotoxic Shiga toxins (Stx), which are responsible for kidney injury and development of hemolytic uremic syndrome. The endoplasmic reticulum (ER) stress response is hypothesized to induce apoptosis contributing to organ injury; however, this process has been described only *in vitro*. ER stress marker transcripts of spliced XBP1 (1.78-fold), HSP40 (4.45-fold) and CHOP (7.69-fold) were up-regulated early in kidneys of Stx2 challenged mice compared to saline controls. Anti-apoptotic Bcl2 decreased (−2.41-fold *vs.* saline) and pro-apoptotic DR5 increased (6.38-fold *vs.* saline) at later time points. Cytoprotective activated protein C (APC) reduced early CHOP expression (−3.3-fold *vs.* untreated), increased later Bcl2 expression (5.8-fold *vs.* untreated), and had early effects on survival but did not alter DR5 expression. Changes in kidney ER stress and apoptotic marker transcripts were observed in Stx2-producing *C. rodentium* challenged mice compared to mice infected with a non-toxigenic control strain. CHOP (4.14-fold) and DR5 (2.81-fold) were increased and Bcl2 (−1.65-fold) was decreased. APC reduced CHOP expression and increased Bcl2 expression, but did not alter mortality. These data indicate that Stx2 induces renal ER stress and apoptosis in murine models of Stx2-induced kidney injury, but decreasing these processes alone was not sufficient to alter survival outcome.

## 1. Introduction

Shiga toxin-producing enterohemorrhagic *Escherichia coli* (EHEC) are a significant cause of food-borne illness in the United States [1]. When the first U.S. outbreak of the EHEC strain O157:H7 was described in 1982, it was considered a rare pathogen [2], but is now an annual public health problem and data from recent outbreaks suggests that more virulent strains have emerged [3]. Infections tend to be seasonal, occurring from May to November, and the majority are due to contaminated ground beef or fresh produce [4]. *E. coli* O157:H7 is the most common infecting strain and has an infectious dose of <100 organisms [5]. Infection presents as a prodromal hemorrhagic colitis [6], and the likelihood that complications will develop is increased in children younger than five years old [7]. An estimated 5%–15% of EHEC infected individuals will develop the potentially lethal complication of hemolytic uremic syndrome [8], which is clinically defined as thrombotic microangiopathy, thrombocytopenia and microangiopathic hemolytic anemia, contributing to acute kidney injury or failure. There is significant morbidity associated with Stx-driven kidney injury, and many patients with diarrhea-associated HUS will have long term renal impairment [9]. The use of antibiotics is contraindicated for O157:H7 EHEC, because their use increases the risk of HUS development [10], potentially by upregulating the pathogen’s toxin expression [11]. As no approved pathogen- or toxin-specific therapies are available, treatment is limited to supportive care.

Production and secretion of the ribosome inactivating Shiga toxins (Stx1, Stx2 and variants) defines EHEC. It is well accepted that these toxins are the pathogen’s primary virulence factor [12], and strains that produce Stx2 are epidemiologically associated with more severe disease [13]. The Shiga toxins are AB_5_ holotoxins, consisting of a pentameric B subunit non-covalently associated with an A subunit [14]. The B subunit mediates toxin binding to its cell surface receptor, Gb_3_, and the A subunit, which has RNA *N*-glycosidase activity, is responsible for the toxic action. Following Stx binding to Gb_3_, the toxin is endocytosed [15], and the A subunit is nicked by furin, generating an A1 fragment that is linked to the A2-B subunit by a disulfide bond [16]. The toxin undergoes retrograde transport to the Golgi apparatus and the endoplasmic reticulum (ER) [17], wherein the disulfide bond is reduced. The now active Stx A subunit translocates to the cytosol, where it cleaves an adenine residue from 28S ribosomal RNA, thus preventing elongation factor 1-dependent peptide chain elongation, leading to cessation of protein synthesis [18].

Though the enzymatic action of Stx is well characterized, less is known about mechanisms that give rise to cell death and organ dysfunction *in vivo*. Stx induces apoptosis in various cell types *in vitro* [19,20], and evidence of renal apoptosis has been observed clinically and in animal models [21]. *In vitro* challenge of monocyte-like THP-1 cells with Stx1 activates ER stress responses, culminating in apoptosis [22]. A clear understanding of how the toxin impacts intracellular pathways in an *in vivo* context, particularly in the kidneys, will provide insight into opportunities for development of non-antibiotic therapeutics.

In order to explore the roles that ER stress and apoptosis play in cell death and kidney dysfunction *in vivo*, we examined expression of cellular ER stress markers, as well as anti- and pro-apoptotic markers in animal models of Stx2-induced kidney injury using either Stx2 challenge alone or Stx2 exposure from intestinal infection with toxin-producing *Citrobacter rodentium* [23]. We identified renal ER stress and apoptosis responses to Stx2 and sought to modulate these using activated protein C (APC), an anti-coagulant and cytoprotective molecule (reviewed in [24]). APC has been shown to be protective against thapsigargin-induced ER stress *in vitro* [25], and protects against the pan-caspase inhibitor Z-VAD-FMK [26]. Our studies reveal novel insights into the mechanism of Stx2-induced cell death and kidney dysfunction *in vivo*.

## 2. Results

### 2.1. Murine Responses in Two Injection Stx2 Model

**Figure 1:**
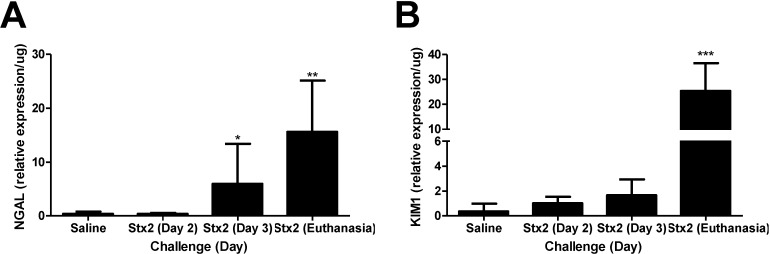
Stx2 challenge increases kidney injury marker expression. Mice were challenged with 0.05 ng Stx2/g body weight (*n* = 4–12) by i.p. injection and were euthanized on either Day 2, Day 3 or upon reaching euthanasia criteria typically by Day 4–6. Control mice (*n* = 5) were challenged with sterile saline and kidneys were harvested by Day 5–10. Mean values ± S.D. are shown. Measurement of kidney injury marker gene transcripts for neutrophil gelatinase-associated lipocalin (NGAL) (**A**) and kidney injury marker 1 (KIM1) (**B**) were quantified after RNA isolation and qPCR. Significance was determined by Kruskal-Wallis test with Dunn’s multiple comparison test. * *p* < 0.05, ** *p* < 0.001, *** *p* < 0.001.

mRNA expression levels of kidney injury markers neutrophil gelatinase-associated lipocalin (NGAL) [27] and kidney injury marker 1 (KIM1) [28] were assessed by qPCR using kidney tissue from mice who received intraperitoneal Stx2 challenge (0.05 ng Stx2/g body weight) on Day 0 and Day 3, or saline (control). This 2-injection design is essentially a lethal toxemia model with ~93% lethality within 4–7 days after first toxin challenge (*n* = 44). Stx2 was utilized because EHEC strains that produce Stx2 are associated with greater disease severity [13,29]. To identify early responses, kidneys were harvested at Day 3 (before the second toxin injection) whereas late responses were measured when animals reached endpoint euthanasia criteria. By Day 3 post-challenge, renal NGAL expression in Stx2 challenged mice was significantly elevated 14.3-fold compared to saline challenged mice (Figure 1A; *p* < 0.05), and by euthanasia, expression was over 30-fold higher (*p* < 0.01). By Day 3, renal KIM1 expression in Stx2 challenged mice was increasing but not statistically different from saline control mice kidneys (4.5-fold higher; n.s.). At euthanasia, renal KIM1 mRNA in toxin challenged animals was considerably elevated relative to controls (69.5-fold; *p* < 0.001) (Figure 1B). Consistent with all published Stx murine models, there were no changes in platelet levels or coagulation indices (data not shown). Taken together, these data demonstrate that renal transcriptional changes consistent with kidney injury occur in Stx2 challenged mice beginning at Day 3 post challenge continuing through euthanasia.

### 2.2. ER Stress and Apoptosis

**Figure 2:**
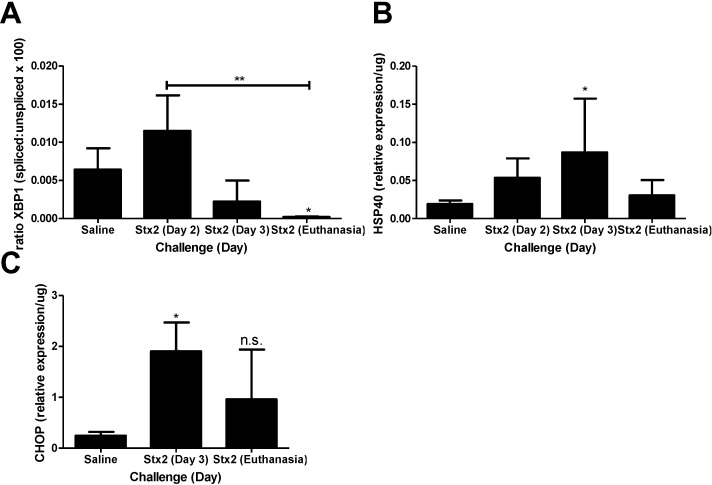
Murine kidney injury following Stx2 challenge is accompanied by ER stress. Mice were challenged with 0.05 ng Stx2/g body weight (*n* = 3–6) by i.p. injection and were euthanized on either Day 2, Day 3, or upon reaching euthanasia criteria typically by Day 4–6. Control mice (*n* = 3–5) were challenged with sterile saline. Kidneys were processed for downstream analysis of ER stress marker transcripts as described in Section 4, Materials and Methods. Mean ± S.D. are shown. (**A**) Measurement of XBP1 mRNA splicing with time; (**B**) Measurement of kidney HSP40 mRNA expression with time; (**C**) Measurement of kidney CHOP expression with time. Significance was determined by Kruskal-Wallis test with Dunn’s multiple comparisons test. Significance is compared to saline. * *p* < 0.05, ** *p* < 0.01, n.s. not significant.

**Figure 3:**
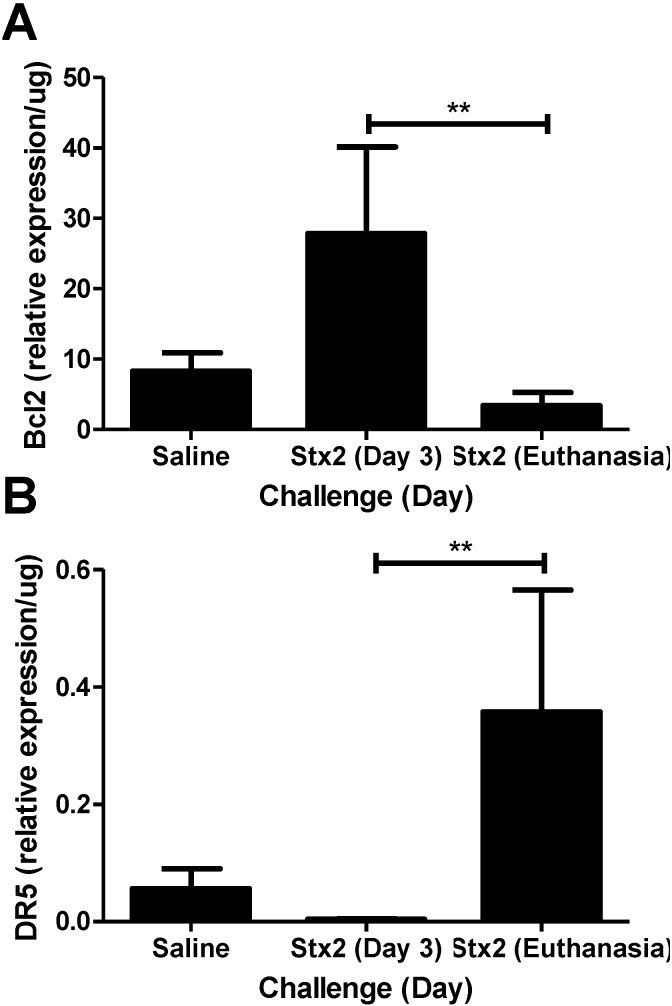
Changes in anti- and pro-apoptotic markers in kidneys. Mice were challenged with 0.05 ng Stx2/g body weight (*n* = 4–6) or saline (*n* = 5) by i.p. injection and were euthanized on either Day 3 or upon reaching euthanasia criteria by Day 4–6. Kidneys were processed for downstream analysis of (**A**) anti-apoptotic marker Bcl2 and (**B**) pro-apoptotic marker DR5 transcripts as described in *Materials and Methods*. Mean ± S.D. are shown. Significance was determined by Kruskal-Wallis test with Dunn’s multiple comparison test. ** *p* < 0.01.

To address the hypothesis that Stx2 induces ER stress and apoptotic processes *in vivo*, kidneys from saline and Stx2 challenged animals were harvested for assessment of relevant pathway mRNA transcripts. Renal gene expression patterns of Stx2 challenged mice were consistent with early ER stress activation (XBP1, HSP40, CHOP) and later changes in apoptosis markers (Bcl2, DR5). IRE1α pathway activation results in spliced XBP1 mRNA which is quantified by PCR and loss of a restriction enzyme site. Compared to saline controls, kidney XBP1 splicing increased 1.78-fold on Day 2 post-challenge, and rapidly decreased at Day 3 and euthanasia (Days 4–6; *p* < 0.05) (Figure 2A). Kidney expression of the BiP co-factor HSP40, which is downstream of spliced XBP1 [30], was increasing on Day 2 (2.79-fold compared to saline control) and significantly elevated on Day 3 (4.54-fold; *p* < 0.05) (Figure 2B). Kidney expression of the ER stress marker CHOP, which is at the intersection of ER stress and apoptosis [31,32], also was up-regulated significantly by Day 3 (7.69-fold compared to saline; *p* < 0.05) and remained high at euthanasia (3.88-fold increase) but differences did not reach statistical significance (Figure 2C). The early (Day 2, 3) ER stress responses in the kidney were accompanied by increasing anti-apoptotic Bcl2 mRNA (3.35-fold compared to saline control) that was significantly reduced at euthanasia (Figure 3A; *p* < 0.01). Coincident with this late change in Bcl2 was considerable up-regulation of pro-apoptotic death receptor 5 (DR5) message from the extrinsic apoptosis pathway at euthanasia (96.9-fold; *p* < 0.01) (Figure 3B).

### 2.3. Activated Protein C and Z-VAD-FMK Treatment

**Figure 4:**
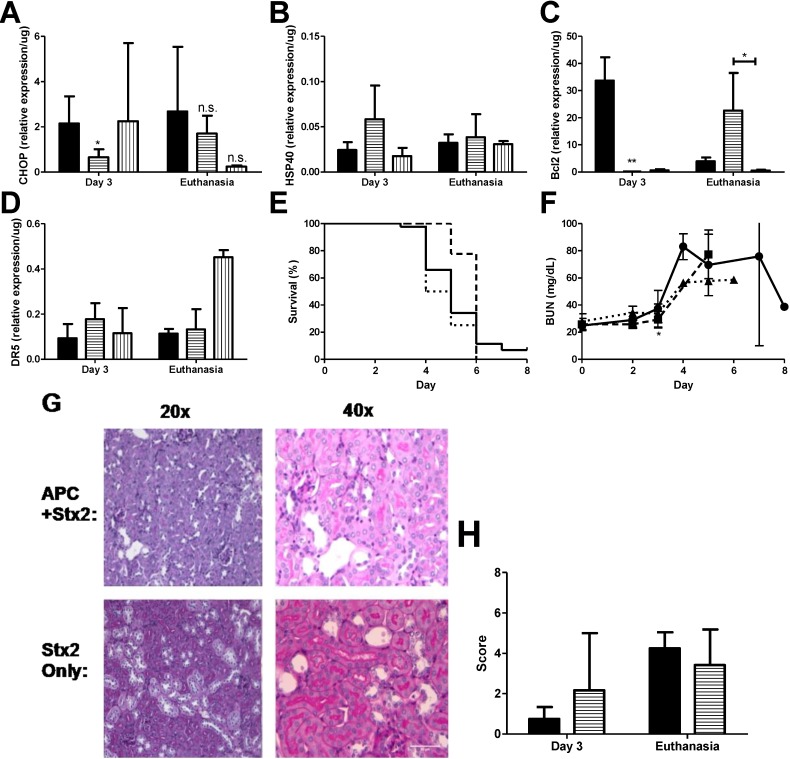
APC treatment reduces early ER stress markers and delays anti-apoptotic patterns. Stx2 challenged mice (solid, ●) were treated with 20 μg activated protein C daily on Days 0–3 (horizontal stripes, ■) or with 125 μg Z-VAD-FMK (vertical stripes, ▲) and were euthanized on either Day 3 or upon reaching euthanasia criteria. Kidneys were harvested and processed for mRNA markers. (**A**) ER stress marker CHOP (*n* = 3–6) and (**B**) HSP40 (*n* = 5); (**C**) Kidney mRNA expression of anti-apoptotic marker Bcl2 (*n* = 5) and (**D**) pro-apoptotic marker DR5 (*n* = 4–5); (**E**) Kaplan-Meier curve of survival of challenged animals (Stx2, solid, *n* = 44; APC + Stx2, dashed, *n* = 10; Z-VAD-FMK + Stx2, dotted, *n* = 4); (**F**) Plasma BUN was measured in challenged animals as a marker of kidney injury; (**G**) PAS stained kidney of challenged animals. Renal tubular epithelial injury predominated and no pathology differences were observed between cortex or medulla tissue. Representative images are shown; (**H**) Histology score of Stx2 challenged animals; *n* = 2 per group with six images per animal. All images were blinded prior to analysis. Significance was determined by either Kruskal Wallis test with Dunn’s multiple comparison test (**A**–**D**) or repeated measures ANOVA with Bonverroni post-test (**F**), and is compared to Stx2 only unless otherwise indicated. * *p* < 0.05, ** *p* < 0.01, n.s. not significant.

Anti-coagulant and cytoprotective APC protects against *in vitro* thapsigargin-induced ER stress and apoptosis in THP-1 cells, as demonstrated by reduction in the levels of both 78-kDa glucose-regulated protein and CHOP, as well inhibition of ER Ca^2+^ flux, [25] which gives rise to the possibility that APC may have similar characteristics *in vivo*. Stx2 challenged mice were treated with 20 μg APC by i.p. injection beginning on Day 0 just before the first toxin injection and continued treatment up to Day 3 as described in *Materials and Methods*. APC dosing was chosen based on pilot studies in the baboon Stx2-induced HUS model (data not shown; [33]). Kidneys were harvested on Day 3 to assess early ER stress-related markers and at euthanasia. Treatment with APC significantly down-regulated early CHOP expression in the kidneys (Day 3 compared to non-treated; *p* < 0.05) (Figure 4A) and remained low at euthanasia. Compared to toxin only, HSP40 message did not change with APC treatment at either time point (Stx2 only: 0.02 ± 0.009/μg *vs.* APC + Stx2: 0.06 ± 0.004/μg, *p* = 0.07 on Day 3; Stx2 only: 0.03 ± 0.01/μg *vs.* APC + Stx2: 0.04 ± 0.03/μg, *p* = 0.63 at euthanasia endpoint) (Figure 4B) nor were changes in renal XBP1 splicing observed (data not shown).

The effect of APC on kidney anti- and pro-apoptotic marker mRNA expression contrasted in a manner dependent on time point and marker analyzed. APC treatment had a large influence on kidney Bcl2 message both on Day 3 and at euthanasia (Figure 4C). Early kidney Bcl2 expression on Day 3 post-challenge was significantly down-regulated (−188.8-fold compared to non-treated animals, *p* < 0.001). This Bcl2 message was then up-regulated 5.8-fold compared to non-treated animals at euthanasia. APC treatment modestly increased DR5 expression on Day 3 (1.9-fold; Figure 4D) but was not different from toxin only at euthanasia.

APC did have an effect on survival (Figure 4E), but the impact was early so that by Day 5 there was 77.8% survival for APC treated mice (dashed line) but only 34.1% survival for the untreated mice (solid line). Overall mean survival did not differ between the two groups (Stx2: 5.02 ± 1.05 days *vs.* APC + Stx2: 5.78 ± 0.44 days). Stx2-induced plasma BUN elevation was delayed significantly in APC treated animals (Figure 4F; *p* < 0.05 on Day 3), but kidney mRNA expression of injury markers NGAL and KIM1 were unchanged at this time point (data not shown). Kidney pathology at the light microscopy level was not impacted by APC treatment and a similar renal tubular injury pattern was observed regardless of treatment (Figure 4G,H).

That renal DR5 expression was unchanged in APC treated Stx2 challenged animals at euthanasia led us to hypothesize that its up-regulation was causing continued apoptotic signaling even during Bcl2 increases. This has been shown for Fas [34] so it remained possible that ligand-independent DR5 signaling via the caspase cascade was important [35]. To determine if pro-apoptotic DR5 is playing a role in the lethal kidney injury, Stx2 challenged mice were treated with the pan-caspase inhibitor Z-VAD-FMK [26] beginning at Day 0 and continuing through Day 3. Autoproteolytic cleavage of initiator caspases 8 and 10 occurs following DR5 mediated recruitment of the death induced signaling complex [35], and these initiator caspases are targets of Z-VAD-FMK. The kidney mRNA expression levels of HSP40, CHOP and DR5 were unchanged by Z-VAD-FMK treatment at any time point analyzed compared to either Stx2 alone or APC + Stx2 (Figure 4A,B,D). Renal Bcl2 expression was decreased on both Day 3 and at euthanasia (*p* < 0.05 compared to APC + Stx2) in Z-VAD-FMK treated mice (Figure 4C). Z-VAD-FMK treatment provided no survival benefit (Figure 4E) and eliminated APC’s survival benefit (data not shown).

### 2.4. ER Stress, Apoptosis, and APC Treatment in Stx2-Producing Citrobacter rodentium Model

Oral gavage and intestinal infection with Stx2-producing *C. rodentium* model of Stx2 provides a reasonable replicate of the time course of human infection with EHEC as well as constant exposure to Stx2 with resultant kidney injury [23]. To confirm the renal transcriptional patterns consistent with ER stress, apoptosis and APC effects observed in the Stx2 toxemia model, kidneys were harvested at euthanasia from mice challenged with ~1 × 10^9^ CFU Stx2 producing *C. rodentium* (C.r + Stx2), with or without APC co-treatment, or non-Stx2 producing *C. rodentium* (C.r-control). Survival, plasma BUN, and kidney mRNA expression patterns were assayed as described in *Materials and Methods*.

**Figure 5:**
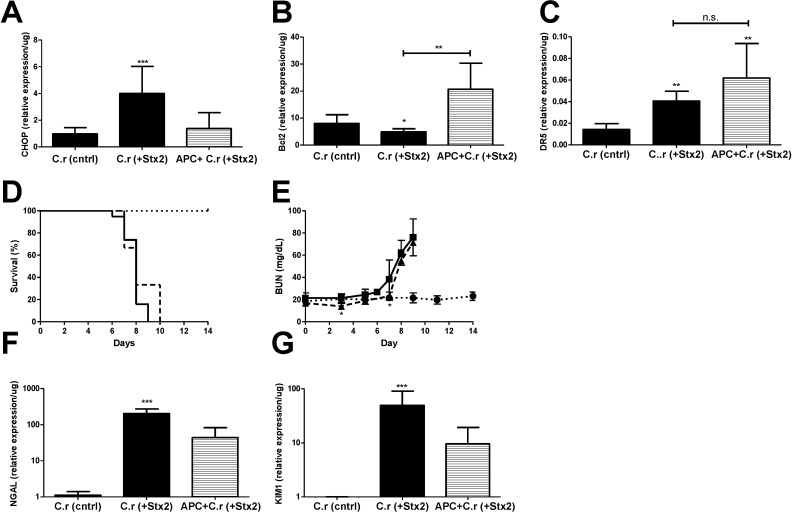
ER stress accompanies Stx2 producing *C. rodentium* induced lethal kidney injury, and is abolished through APC co-treatment. Mice were challenged with ~1 × 10^9^ CFU of Stx2 producing *Citrobacter rodentium* (*n* = 9–19; solid, ■) either alone or with 20 μg APC treatment (*n* = 3; dashed, ▲). Control mice were challenged with non-toxigenic *C. rodentium* (*n* = 10; dotted, ●). Means ± S.D. are shown. (**A**) Kidney mRNA expression of ER stress marker CHOP; (**B**) anti-apoptotic Bcl2 and (**C**) pro-apoptotic marker DR5; (**D**) Kaplan-Meier analysis of survival; (**E**) Plasma BUN was measured with time in challenged animals as a marker of kidney injury; (**F**–**G**) Kidney mRNA expression of injury markers NGAL (**F**) and KIM1 (**G**). Significance was determined by Kruskal-Wallis test with Dunn’s multiple comparison test (**A**–**C**,**F**,**G**) or repeated measures ANOVA with Bonferroni post-test (**E**) and is compared to C.r-control unless otherwise specified. * *p* < 0.05, ** *p* < 0.01, *** *p* < 0.001, n.s. not significant.

Kidney CHOP expression at euthanasia in C.r + Stx2 challenged animals was elevated significantly (4.14-fold compared to C.r-control; *p* < 0.001) and was reduced in APC treated animals but did not reach statistical significance (−2.92-fold compared to non-treated; n.s.) (Figure 5A). Renal anti-apoptotic Bcl2 was significantly decreased in C.r + Stx2 challenged animals (−1.65-fold compared to C.r-control, *p* < 0.05) and APC treatment increased its expression (4.24-fold compared to C.r + Stx2, *p* < 0.001) (Figure 5B). Pro-apoptotic DR5 was up-regulated significantly (2.81-fold compared to C.r-control, *p* < 0.01) in C.r- + Stx2 challenged animal (Figure 5C). APC treatment had no effect on renal DR5 expression (Figure 5C), which remained significantly elevated compared to control animals (C.r + Stx2 0.04 ± 0.01/μg *vs.* C.r-control 0.01 ± 0.01/μg; *p* < 0.01).

APC treatment beginning on Day 0 had early, but modest, effects on plasma BUN and there was no overall survival benefit. Mean survival time of C.r + Stx2 challenged animals (Figure 5D) was the same despite three days of APC treatment (7.8 ± 0.77 days *vs.* 8.3 ± 1.53 days with APC). Plasma BUN (Figure 5E) was significantly lower in APC treated animals as compared to non-treated animals on Day 3 (*p* < 0.05) and Day 7 (*p* < 0.05), but the differences were biologically small. APC treatment also had little or no effect on kidney mRNA expression of kidney injury markers NGAL or KIM1 compared to un-treated animals (Figure 5F,G). Comparatively, APC treatment impacted the Stx2 injection model to a greater extent than the Stx2-producing *C. rodentium* model, which may be due to timing and duration of Stx2 exposure in the host.

## 3. Discussion

### 3.1. Stx2-Induced Murine Lethal Kidney Injury Is Accompanied by Transcriptional Evidence of ER Stress and Apoptosis

The studies presented show, for the first time, that Stx2 induced lethal kidney injury *in vivo* is accompanied by renal transcriptional changes in ER stress and apoptotic markers. Transcriptional alterations were observed in two models of Stx2 challenge, both of which developed significant kidney injury. Lee *et al.* showed that Stx1 challenge of THP-1 cells results in caspase-8 activation, leading to processes consistent with the formation of the apoptosome [36], and ultimately identified the mechanism responsible for the observed apoptosis as being the ER stress response [22]. Our work extends these observations in that many of the ER stress markers identified as up-regulated following *in vitro* Stx1 challenge are similarly up-regulated in the kidneys of *in vivo* models of Stx2 induced kidney injury, including increased XBP1 splicing and up-regulated CHOP, which is at the point in the ER stress pathway where it switches from a homeostasis restoration effort to a programmed cell death effort [31,32].

An interesting finding of this study was the characterization of the timing of transcriptional alterations in anti-apoptotic Bcl2 and pro-apoptotic DR5. The finding that the renal transcriptional pattern is strongly anti-apoptotic on Day 3 post-challenge is novel, and supports an anti-apoptotic function for the renal ER stress response early in Stx2 induced kidney pathogenesis. This hypothesis is further supported by the finding that APC co-treatment resulted in opposite expression patterns of anti-apoptotic Bcl2 (down-regulated) and pro-apoptotic DR5 (up-regulated) on Day 3. The renal anti-apoptotic transcriptional pattern coincident with renal transcriptional changes consistent with ER stress on Day 3 post-Stx2 challenge suggests that the Stx2-induced ER stress may thus be an adaptive response that serves a cytoprotective purpose. We observed that kidney mRNA expression of anti-apoptotic Bcl2 and pro-apoptotic DR5 were down-regulated and up-regulated, respectively at euthanasia; these observations are consistent with DNA fragmentation following Stx1 or Stx2 challenge of human renal cortical epithelial cells [19], and dUTP nick end labeled cells in renal cortices of EHEC infected mice and children with HUS [21].

### 3.2. APC Treatment of Stx2 and Stx2 Producing C. rodentium Challenged Mice Down-Regulates ER Stress but Does Not Reverse Lethal Kidney Injury

In order to address the hypothesis that Stx2-induced ER stress and apoptosis are driving mechanisms toward kidney cell death and organ injury, we treated mice with APC, an anti-coagulant with described cytoprotective effects, that has been shown to down-regulate thapsigargin induced ER stress processes *in vitro* [25]. Though the molecular mechanisms mediating APC’s protective effects on ER stress pathways are unknown, *in vitro* APC treatment has previously been demonstrated to reduce NFκB activity and to down-regulate proapoptotic p53 and Bax expression (reviewed in [24]). If ER stress and downstream apoptotic processes are playing major roles in Stx2 induced organ failure, then down-regulation of these processes should reverse the transcriptional evidence of these processes and rescue Stx2 challenged mice. Consistent with described *in vitro* studies, mice treated with APC showed statistically significant decreases in kidney CHOP mRNA as well as increased expression of anti-apoptotic Bcl2 at euthanasia. APC’s effects on ER stress and apoptosis transcripts were observed in both models of Stx2 toxemia, whether delivered directly by injection or by more natural daily exposure from the intestinal bacterial infection. These data extend the *in vitro* observations of Toltl *et al.* [25] and show that APC can down-regulate ER stress *in vivo*. However, these changes in ER stress markers were not accompanied by changes in either renal DR5 expression or in kidney injury marker expression, and were not able to prevent mortality in either model.

### 3.3. Z-VAD-FMK Treatment of Stx2 Challenged Mice Is Ineffective

These data led us to hypothesize that, despite the renal Bcl2 up-regulation observed in APC treated mice, the unchanged kidney DR5 expression might allow for continued apoptotic signaling in the kidneys of these animals. This hypothesis is consistent with the work of Huang *et al.*, who demonstrated that up-regulation of anti-apoptotic Bcl2 and the related anti-apoptotic Bcl-x_L_ is unable to rescue murine primary cells from apoptotic signaling mediated by the DR5-related pro-apoptotic Fas [34]. However, co-treatment of Stx2 challenged mice with the pan-caspase inhibitor Z-VAD-FMK, either alone or in conjunction with APC, was unable to rescue animals from lethal kidney injury and actually ameliorated APC’s alterations of kidney transcriptional patterns and modest survival benefit.

### 3.4. Targeting Intracellular Stx2 Activities Remains a Viable Therapeutic Opportunity

Taken together, the current data do not support a major role for ER stress and/or apoptotic processes in Stx2-induced kidney organ injury leading to organ failure, suggesting that these pathways are likely not reasonable therapeutic targets. However, when taken with observations made by others, the current study adds insight into the intracellular Stx2 activities that contribute to toxin-induced cell death. Retrograde transport from cell membrane to the ER for toxic activity is shared by many AB_5_ holotoxins [37,38] and brefeldin A inhibits the formation of functional Golgi complexes to abolish Stx1-induced DNA fragmentation in THP-1 cells [39]. Nishikawa *et al.* and Stearns-Kurosawa *et al.* demonstrated that EHEC challenged mice [40] and Stx2 challenged non-human primates [41] can be rescued by administration of a therapeutic cell permeable peptide, and the mechanism of rescue was related to the intracellular trafficking of Stx2 [40]. Further highlighting the importance of trafficking to the ER in Stx cytoxicity is the work of Smith *et al.*, who described a requirement for adequate glucosylceramide cellular levels for appropriate Stx1 trafficking [42]. Under low glucosylceramide conditions, Stx1 binds its receptor and is trafficked to the ER, but it is no longer able to translocate across the ER membrane and cytotoxicity is abolished. Thus, although down-regulation of Stx2-induced ER stress responses is unable to prevent renal kidney cytotoxicity and loss of function (at least in mice), Stx2 trafficking to the ER remains an essential step in Stx2-mediated cell death and a potential therapeutic target.

## 4. Materials and Methods

### 4.1. Reagents

Shiga toxin 2 was purchased from the Phoenix Lab (Tufts University Medical Center, Boston, MA, USA). Endotoxin contamination was minimized by incubation with polymixin B-agarose beads (Sigma, St. Louis, MO, USA) and confirmed with the Pierce LAL chromogenic endotoxin quantitation kit (Thermo Scientific, Rockford, IL, USA). Plasma blood urea nitrogen (BUN) was assayed using the QuantiChrom Urea Assay Kit (BioAssay Systems, Hayward, CA, USA).

### 4.2. Citrobacter rodentium Culture

*C. rodentium* strains DBS770 (λ*stx_2dact_*) and DBS771 (λ*stx_2dact_::kan^R^*), which are Gram-negative rodent enteropathogens, were kindly provided by John M. Leong (Department of Molecular Biology and Microbiology, Tufts University Medical Center, Boston, MA, USA, [23]). Shiga toxin producing *C. rodentium* was lysogenized to express Stx2 (C.r + Stx2, DBS700); non-Shiga toxin producing *C. rodentium* was lysogenized to express Stx2, with a kanamycin resistance marker inserted within an in-frame deletion of the Stx2 gene (C.r-control, DBS771). Bacteria were grown in LB broth with either chloramphenicol (10 mg/mL) only (C.r + Stx2) or both chloramphenicol (10 mg/mL) and kanamycin (25 µg/mL) (C.r-control). Dosing concentrations for oral gavage were quantified from a pre-determined standard curve (OD600 *vs.* CFU) and confirmed by standard plating methods.

### 4.3. Animal Experiments

Six week old C57Bl/6J mice were purchased from The Jackson Laboratory (Bar Harbor, ME, USA). All animal experiments were approved by the BUMC Institutional Animal Care and Use Committee. Mice were housed under a 12-h light-dark cycle and allowed access to standard diet and water *ad libitum*. For the injected Stx2 model, male mice were used, and groups were challenged with either 0.05 ng Stx2/g body weight or sterile saline by intraperitoneal (I.P.) injection on Days 0 and 3. Stx2 dose was chosen based on pilot experiments measuring dose responses to single and multiple injections (data not shown). Animals were weighed and observed daily and phlebotomy was performed periodically. Animals were euthanized on Days 2 or 3 (before the second toxin injection) or upon reaching pre-defined euthanasia criteria. For activated protein C (APC) treatment experiments, animals were challenged as described above, with 20 µg APC (kindly provided by Kaketsuken, Kumamoto, Japan) given by daily IP injection on Days 0–3, with or without 125 µg Z-VAD-FMK (Bachem, Torrence, CA, USA). For *C. rodentium* experiments, female mice were used. Groups were challenged by oral gavage with 0.9–1.2 × 10^9^ CFU *C. rodentium* either alone or with 20 µg APC by IP injection daily through Day 5, the longer dosing period to allow for continued intestinal colonization and toxin exposure. Animals were weighed and observed daily with periodic phlebotomy with occasional feces collection for colonization confirmation (data not shown). For all experiments, organs were collected at necropsy and were either flash frozen or stored in RNAlater (Ambion, Austin, TX, USA) or 10% neutral buffered formalin for downstream processing.

### 4.4. RNA Isolation and qPCR

Tissues stored in RNAlater were thawed on ice and aseptically dissected. Tissue was lysed with a 5 mm bead in the Tissue Lyser II for 4 min at 25 Hz (Qiagen, Hilden, Germany) in the presence of buffer RLT plus with 1% beta-mercaptoethanol (Qiagen). RNA extraction from tissue lysate was performed using the RNeasy plus mini kit following manufacturer’s instructions. RNA concentration was quantified using the Nano Drop Spectrophotometer (Thermo Scientific).

Total RNA was made into cDNA using the Quantifast RT kit (Qiagen) and Thermocycler (Applied Biosystems, Beverly, MA, USA) according to manufacturer’s instructions. Either 250 ng or 2 μg RNA was used for each reverse transcription reaction.

Amplification of cDNA was performed in a Step One Plus qPCR machine (Applied Biosystems) using the Quantifast SYBR green PCR kit (Qiagen) according to manufacturer’s instructions and 1 μmol/L of the appropriate forward and reverse primer sets (Table 1). Each sample was analyzed in duplicate. Obtained CT values were normalized as follows: [(2^CT_gene_)/(2^CT_housekeeper_)]/[total RNA in RT reaction].

**Table 1:** Forward and reverse primer pairs.

Gene	Sequence
*Lcn1* (NGAL)	F: 5'CCCTGTATGGAAGAACCAAGGA3'
	R: 5'CGGTGGGGACAGAGAAGATG3'
*Kim1*	F: 5'GGAGATACCTGGAGTAATCACACTG3'
	R: 5'TAGCCACGGTGCTCACAAGC3'
*Xbp1*	F: 5'AAACAGAGTAGCAGCTCAGACTGC3'
	R: 3'ATCTCTAAGACTAGGGGCTTGGT3'
*ERdj4* (HSP40)	F: 5'AGGAACCTGGGAGCTTGACTA3'
	R: 5'ACACATGACGTGCTTGGAATG3'
*Bcl2*	F: 5'TTCTTTGAGTTCGGTGGGGTC3'
	R: 5'TGCATATTTGTTTGGGGCAGG3'
*Dr5*	F: 5'TTCCAGTAGTGCTGCTGATTGG3'
	R: 5'CAAACGCACTGAGATCCTCCT3'
*Ddit3* (CHOP)	F: 5'AGTTATCTTGAGCCTAACACGTCG3'
	R: 5'CACTTCCTTCTGGAACACTCTCTC3'
*Hprt*	F: 5'TGGGCTTACCTCACTGCTTTC3'
	R: 5'CCTGGTTCATCATCGCTAATCAC3'

### 4.5. Spliced XBP1 Assay

cDNA was prepared from 2 µg kidney RNA as described above, and amplification was performed in a Thermocycler (Applied Biosystems) using the Taq PCR master mix (Qiagen) according to manufacturer’s instructions and 0.3 µmol/L of XBP1 forward and reverse primers (Table 1). In each cycle, denaturing was at 95 °C for 45 s, annealing at 55 °C for 45 s, extension at 72 °C for 45 s and a final extension at 72 °C for 4 min. A 26 base pair fragment containing a PstI restriction site is removed following XBP1 mRNA splicing. PCR products were digested with 20 units PstI (Thermo Scientific) at 37 °C for 1 h. Digested PCR products were mixed with a loading dye (Qiagen) and were resolved on a 2% agarose gel containing the SYBR safe DNA gel stain (Life Technologies, Carlsbad, CA, USA). Microdensitometry plots of spliced/unspliced XBP1 bands and housekeeper gene bands were created using ImageJ software (NIH, Bethesda, MD, USA), and the ratio of spliced to unspliced XBP1 was normalized to the housekeeper gene.

### 4.6. Histology

Slides were prepared and PAS stains were performed by the Histology core in the Department of Pathology and Laboratory Medicine at the Boston University School of Medicine. Representative images are shown. Two animals were analyzed per group, and six images were analyzed per animal. All images were blinded before analysis and scoring is described in Table 2.

**Table 2:** Scoring criteria to quantify kidney damage observed in PAS stained images.

Criteria (6 fields analyzed per animal)	Score	
Injury foci in low magnification field (20×)	0	0
	1 per field	1
	2 per field	1
	3 per field	1
	4+ per field	1
Tubule dilation	0 per field	0
	1–4 per field	1
	4+ per field	1
Epithelial cell shedding	1	
Pyknotic bodies	1	
Total possible Score	8	
